# Perinatal Outcomes of Immigrant Mothers and Their Infants Born Very Preterm across Germany

**DOI:** 10.3390/healthcare12121211

**Published:** 2024-06-17

**Authors:** Britta M. Hüning, Julia Jaekel, Nils Jaekel, Wolfgang Göpel, Egbert Herting, Ursula Felderhoff-Müser, Juliane Spiegler, Christoph Härtel

**Affiliations:** 1 Department of Paediatrics I, Neonatology, Paediatric Intensive Care, Paediatric Neurology, University Hospital Essen, University of Duisburg-Essen, 45147 Essen, Germany; britta.huening@uk-essen.de (B.M.H.); ursula.felderhoff@uk-essen.de (U.F.-M.); 2 Center of Translational Neuro- and Behavioural Sciences, C-TNBS, Faculty of Medicine, University of Duisburg-Essen, 45147 Essen, Germany; 3 Faculty of Education and Psychology, University of Oulu, 90570 Oulu, Finland; nils.jaekel@hum.ku.dk; 4 Department of Psychology, University of Warwick, Coventry CV4 7AL, UK; spiegler_j@ukw.de; 5 Public Health Unit, Finnish Institute for Health and Welfare (THL), 00271 Helsinki, Finland; 6 Department of Psychology, University of Copenhagen, DK-1165 Copenhagen, Denmark; 7 Department of English, German and Romance Studies, Faculty of Humanities, University of Copenhagen, DK-2300 Copenhagen, Denmark; 8 Department of Pediatrics, University Hospital Lübeck, 23538 Lübeck, Germany; wolfgang.goepel@uksh.de (W.G.); egbert.herting@uksh.de (E.H.); 9 Department of Pediatrics, University Hospital Würzburg, 97080 Würzburg, Germany; haertel_c1@ukw.de

**Keywords:** very preterm birth, perinatal and neonatal health, immigrants, language barriers, inequalities

## Abstract

Background: In Germany, more than 40% of infants are born to immigrant parents. Increased survival rates of very preterm (below 32 weeks gestation at birth; VP) infants have not resulted in equally improved life chances and quality of life. More information on perinatal variations in outcomes according to social inequalities, migration background, and language barriers is needed. We tested whether mothers’ immigrant status and language barriers are associated with perinatal health and short-term neonatal outcomes. Methods: The data are from the national multi-centre German Neonatal Network (GNN) cohort, including VP births from 2009 onwards. In total, 3606 (*n* = 1738 female) children were assessed, and 919 (*n* = 449 female) of these children had immigrant backgrounds. Immigrant status was operationalised as a binary variable based on the children’s mothers’ countries of birth (born in Germany vs. foreign-born). Self-reported home language (L1) was used to calculate the average linguistic distance to German as one continuous variable. Results: Mixed-effects models showed that two out of fourteen effects of interest survived the adjustment for known confounders and accounting for the nestedness of data within birth hospitals. Linguistic distance from mothers’ L1s to German was independently associated with diagnoses of preeclampsia (OR = 1.01, 95% CI = [1.00, 1.01]). Infants of foreign-born mothers had higher odds for amniotic infection syndrome (AIS; OR = 1.45 [1.13, 1.86]) than infants of German mothers. Conclusions: Our findings from this large multi-centre longitudinal cohort of VP-born children indicate that maternal immigrant status and language barriers have limited impact on perinatal health and severe neonatal outcomes. This suggests that, regardless of background or language skills, there may be few inequalities in the perinatal health of pregnant women and their newborn preterm infants.

## 1. Introduction

Globally, more than 13 million infants (>10% of all births) are born preterm (<37 weeks of gestational age (GA)) every year [1,2]. In Germany, 8.4% of all infants are born preterm [3], while 1–2% of all infants are born very preterm (VP, <32 weeks of GA) [1]. With improvements in neonatal care, the survival rate of infants at the highest risk for developmental impairment, those born VP, continues to increase. However, increased survival has not resulted in equally improved life chances and quality of life [4,5]. The developmental outcome of very preterm infants is influenced by numerous factors, including biological factors such as immaturity, brain damage/alterations, and medical complications, as well as environmental and social factors [6]. More information on perinatal variations in outcomes related to social inequalities, migration background, and language barriers is needed.

Studies have shown that perinatal outcomes vary between European regions and centres among countries with comparable populations and access to health care [7]. For instance, Pedersen et al. demonstrated that immigrant women in Denmark had an increased risk of small-for-gestational-age and preterm birth [8]. The reasons for these findings are multifaceted and include, for example, maternal length of residence in the country, lifestyle factors, and social inequalities [7,8]. A systematic review by Gagnon et al. reported that immigrants had similar or better outcomes than mothers in their host country with regard to preterm birth, low birth weight, and health-promoting behaviours [9]. A more recent review pointed to complex disparities according to racial and/or ethnic background with regard to neonatal outcomes [10]. In a nationally representative study from the Netherlands, Vidiella-Martin et al. demonstrated a lower risk of mortality during the first year of life among infants born extremely preterm with maternal migration background [11]. In contrast, Rasmussen et al. found complex and severe disparities according to ethnicity and immigrant status with regard to stillbirth and infant death rates among women living in Denmark [12]. Inequalities in maternal levels of education and household income partly explained these findings, while the authors argued that an observed intergenerational risk disproved possible language barrier effects in their sample [12]. Another population-based register study from Denmark pointed to substantial variations in caesarean sections versus vaginal delivery between immigrants and Danish women, with the risk ratio of planned caesarean sections being lower among the majority of immigrant groups [13]. Apart from this, the underlying causes for why certain groups of immigrant women and their infants may be at higher or lower risk for certain adverse outcomes have not been clearly identified.

**Figure 1:**
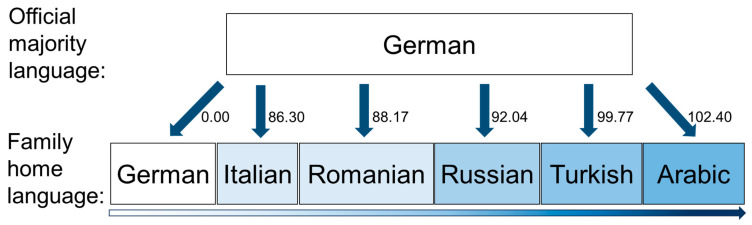
Linguistic distance variable scores among German and five selected immigrant languages.

In 2020, 281 million people were on the move worldwide, an increase of 62% from 2000 [14]. Europe accounts for the largest rise in immigrants [15]. In Germany, almost one in two children (>40%) are born into families with at least one immigrant parent today [16,17], and they are more often growing up with socio-economic inequalities than German children [18]. Migration status is often defined geographically, without considering factors such as legal status, length of stay in the host country, and language barriers. This neglects substantial variations between different individual and societal factors that are associated with access barriers to healthcare [19,20]. For instance, systemic language barriers pose serious challenges to equity and equality of health care [21,22]. Immigrants’ proficiency in a country’s official majority language correlates with health care utilisation [23,24,25] and overall health [26,27]. Speakers of other languages experience different levels of barriers depending on the degree of similarity with their mother tongue (L1). To better operationalise such variations in language barriers, we propose to assess the role of linguistic distance (LD) between mothers’ L1s and the official language of the host country, German. High LD, i.e., greater distance between two languages, has been associated with poor health [19]. In Germany, LD, for example, indicates the level of similarities and differences between Arabic, Turkish, Russian, Romanian, Italian, and German. Turkish L1 mothers, for example, need to overcome a larger LD to the majority language German (LD = 99.77 points) than Romanian L1 mothers in Germany (LD = 88.17 points) (Figure 1).

This study aims to evaluate the association between perinatal health and short-term neonatal outcomes with mothers’ and infants’ immigrant backgrounds and the linguistic distance of their mother tongue (L1) from the official language of the host country, German.

We tested the following hypotheses:

(1) Immigrant status and LD of mothers’ L1s to German are associated with perinatal health and short-term neonatal outcomes, including mode of delivery, complete administration of antenatal corticosteroids for foetal lung maturity, diagnoses of preeclampsia, Haemolysis Elevated Liver enzymes and Low Platelets (HELLP) syndrome, amniotic infection syndrome (AIS), higher grade (>II) intraventricular haemorrhage (IVH), bronchopulmonary dysplasia (BPD), blood-culture proven sepsis, and the total length of hospital stay.
(2) The effects remain stable after adjusting for maternal age and level of education, infant sex, gestation at birth, and multiple birth status, and accounting for the nestedness of data within birth hospitals.

## 2. Materials and Methods

The data are from the national multi-centre observational German Neonatal Network (GNN) cohort study, including all births between 22 + 0 and 31 + 6 weeks of GA from the 1st of April 2009 to the 31st of December 2016 in 68 German neonatal intensive care units (NICU) [28].

### 2.1. Procedures

After obtaining written informed consent from parents or legal guardians, predefined data on general neonatal characteristics, antenatal and postnatal treatments, and outcomes were recorded for each patient. After discharge, data sheets were sent to the study centre (University of Lübeck). A physician or study nurse from the central GNN study office (University of Lübeck) with expertise in neonatology monitored the data quality of the completed record files through annual on-site visits.

**Figure 2:**
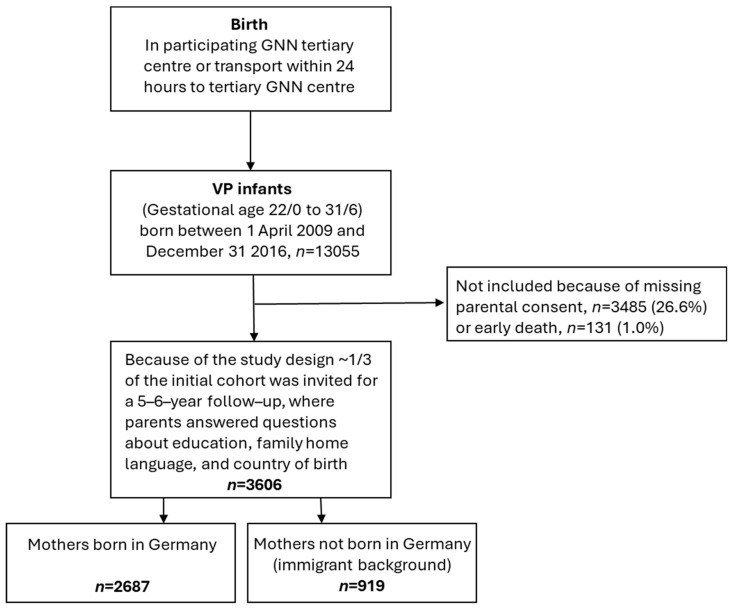
Study design flow chart of the GNN sample assessed to 5–6 years.

At the age of 5–6 years, before children entered formal schooling in Germany, families were invited to the GNN follow-up investigation at each respective study site, where parents answered questions about language use at home. Because of funding constraints, the study design at follow-up included an assessment of only ~30% of the original GNN study participants [28]. A total of 3606 children were followed up; 919 of these children had immigrant background. Figure 2 provides an overview.

All study parts were approved by the University of Lübeck Ethical Committee and the committees of the participating centres (vote no. 08–022). Informed consent was obtained from all subjects. All methods were carried out in accordance with relevant guidelines and regulations, specifically, the Declaration of Helsinki, the current revision of ICH Topic E6, the Guidelines for Good Clinical Practice, and the Guidelines of the Council for International Organization of Medical Sciences, the WHO (“Proposed International Guidelines for Biomedical Research Involving Human Subjects”).

### 2.2. Measures

**Biological and clinical variables.** Information on mothers’ and infants’ biological and clinical characteristics was collected from medical records. This included mothers’ age at birth (years), mode of delivery (vaginal birth, planned caesarean, or emergency caesarean section), diagnoses of preeclampsia, HELLP, and complete administration of antenatal corticosteroids for foetal lung maturity. For infants, this included biological sex, gestational age at birth, birth weight, type of pregnancy (singleton vs. multiple), diagnoses of higher grade (>II) IVH, BPD (defined as any supplemental oxygen or respiratory support at a gestational age of 36 weeks), AIS defined as a combination of clinical signs including maternal fever (≥38.0 °C), foetal or maternal tachycardia, painful uterus and foul-smelling liquor, and laboratory markers such as increased maternal inflammatory markers without any other cause (CRP > 10 mg/L or elevation of white blood cell count > 16,000/μL) and declared as the most likely cause of preterm delivery by the attending obstetric team, blood-culture proven sepsis (defined as clinical sepsis confirmed by the presence of a causative agent in the blood culture or detection in a multiplex real-time PCR), and the total length of hospital stay (in days).

**Mothers’ education**. Information on mothers’ educational qualifications was collected via questionnaires at the 5–6-year follow-up and binary coded into having obtained a high-school degree (German Abitur) vs. not having a high-school degree.

**Immigrant status and linguistic distance.** Immigrant status was operationalised as a binary variable based on mothers’ countries of birth (born in Germany vs. foreign-born [29]). Mothers’ L1s were assessed via questionnaires at the 5–6-year follow-up. Response options consisted of an optional box to tick for “German” and several free-form fields for other languages to be filled. This information was used to calculate the average LD to German as one continuous variable (range 0–103.73, see Appendix A for details). LD was operationalised as one continuous score for each mother according to the Automated Similarity Judgement Program (ASJP) [30]. The LD calculated by the ASJP is based on 40 universally important, culturally independent everyday words and based on the normalised Levenshtein distance [31], i.e., the number of changes, including deletions, insertions, or substitutions, required to transform the phonetic representation of a word from one language to another. For example, water (English) to Wasser (German) has a Levenshtein distance of 3, whereas water (English) to su (Turkish) has a Levenshtein distance of 7. For a detailed discussion of the statistical procedures employed in the calculation of the ASJP LD score, please see [32].

### 2.3. Analysis Strategy

Descriptive analyses and mixed-effects linear or logistic regressions (depending on the scaling of the dependent variables) were carried out in Stata version 17.0. First, to separately test the univariate associations of mothers’ immigrant status and LD with perinatal health and short-term neonatal outcomes, we ran sets of two-level models (i.e., level 1: individuals; level 2: birth hospitals) for each dependent variable, one including a fixed effect of immigrant status, the other including a fixed effect of mothers’ LDs from their L1 to German. Next, for those models that showed significant univariate effects of immigrant status and/or LD, we added fixed effects of maternal age and level of education, infant sex, gestation at birth, and multiple birth status to adjust for known confounders.

## 3. Results

Table 1 outlines descriptive information comparing German (*n* = 2687) vs. foreign-born mothers (*n* = 919). On average, foreign-born mothers were older and less likely to have a high-school degree than German mothers. As expected, most German mothers spoke German as their mother tongue (median LD = 0.00, see Appendix A), while there was a large variation in LD scores among foreign-born mothers. Among the diverse language groups represented in the sample, the largest were Russian, Turkish, English, and Arabic. Foreign-born mothers received fewer diagnoses of HELLP and their infants were born at lower gestational age, were less often multiples, were more often diagnosed with AIS, and spent more days in hospital than infants of German mothers.

**Unadjusted effects of immigrant status.** Fixed effects in two-level mixed models showed that mothers’ foreign country of birth was associated with lower odds for diagnoses of HELLP (*odds ratio* (*OR*) = 0.65 [*95% confidence interval*: 0.48, 0.87], *p* = 0.004), higher odds for diagnoses of AIS (*OR* = 1.60 [1.34, 1.91], *p* < 0.001), and a longer duration of infants’ stays in hospital (3.31 [0.73, 5.88], *p* = 0.012). Mothers’ foreign country of birth was not associated with mode of delivery, preeclampsia, sepsis, complete lung maturity injections, or the frequency of BPD or IVH.

**Unadjusted effects of LD.** Fixed effects in two-level mixed models showed that higher LD to German was associated with lower odds for planned caesarean sections (*OR* per *SD* increase in LD = 0.99 [0.99, 1.00], *p* = 0.037), lower odds of HELLP (*OR* = 0.99 [0.99, 1.00], *p* = 0.035), higher odds of preeclampsia (*OR* = 1.00 [1.00, 1.01], *p* = 0.007), AIS (*OR* = 1.00 [1.00, 0.01], *p* < 0.001), and a longer duration of infants’ stay in hospital (0.05 [0.02, 0.08], *p* = 0.003). LD was not associated with the frequency of emergency caesarean sections, sepsis, lung maturity injections, or BPD or IVH.

**Fully adjusted models**. Two effects of interest remained after adding known confounders to the fully adjusted final models (Table 2 and Table 3). A higher LD from mothers’ L1s to German was independently associated with higher odds for diagnoses of preeclampsia (*OR* = 1.01 [1.00, 1.01]). In this model, if all other factors were held stable, a 1 SD higher LD (i.e., 34.28 points for the total sample) would result in 1.01 higher odds for preeclampsia. Accordingly, for example, the odds for preeclampsia would be on average 1.03 times higher among Arabic-speaking mothers living in Germany (LD = 102.04, *n* = 35) compared with German-speaking mothers. In addition, infants of foreign-born mothers had higher odds for AIS (*OR* = 1.45 [1.13, 1.86]) than infants of German mothers. Random effects indicated variation in associations according to birth hospitals.

## 4. Discussion

This study shows that maternal immigrant background and language barriers have a limited impact on perinatal health and neonatal outcomes in this sample. After adjusting for possible confounders, the only remaining associations were increased odds for incidence of AIS (among foreign-born mothers and their infants) and preeclampsia (among mothers with higher LD). Our findings from this large, German-wide, longitudinal GNN cohort of children born VP therefore suggest that there may be only marginal inequalities in the care for pregnant women and their preterm infants, irrespective of their countries of origin or language proficiency.

The majority of previous studies examining the effect of immigrant status on perinatal outcome investigated mode of delivery, birth weight, or infant mortality [33]. No differences were found in the mode of delivery in our cohort, including emergency caesarean section, or birth weight, but the evidence is inconclusive and varied between migrant women’s countries of origin and the host country. As we only analysed a subsample of the original cohort, specifically those infants who survived and attended the 5-year follow-up, we were not able to investigate infant mortality. However, this study provides reassurance that severe neonatal morbidity, including intraventricular haemorrhage (IVH) and bronchopulmonary dysplasia (BPD), both important for neurodevelopmental outcomes, did not substantially differ between groups or according to linguistic distance. This may be partly due to the fact that the administration of antenatal corticosteroids for lung maturation was carried out to the same extent in foreign-born mothers as in German-born mothers, indirectly indicating that the majority of mothers received timely antenatal care.

In the unadjusted analyses, infants of foreign-born mothers and those with larger LD had longer hospital stays. Longer hospital stays can occur because of complications or as a result of family-centred parent counselling, which is an integral part of many centres in Germany. The Joint Federal Committee mandates this care in the quality guidelines [34]. Parent counselling prepares parents for the discharge of premature babies, improves their skills through training, and ensures a smooth transition home by organizing ambulatory care [35,36]. When language barriers exist, the process may take longer, requiring repeated training courses and additional support services to be arranged for the time at home. However, this association did not remain when controlling for known confounders.

One of the two effects of interest that remained after the addition of known confounders to the fully adjusted final models was a higher incidence of AIS in foreign-born mothers. A higher rate of infection was also reported in migrant mothers in 63.6% of the studies included in a systematic review by Gagnon et al. [9]. It is important to note that in this study, all maternal and paediatric infections, including HIV, toxoplasmosis, etc., were counted as infections and that the data did not explicitly identify an AIS. AIS refers to an infection and/or inflammation of the amnion and placenta. It is usually caused by microbial invasion of the amniotic cavity, either before or after premature rupture of membranes. Intra-amniotic inflammation (IAI) is a serious complication that can have both short and long-term consequences for the child and may be clinically inapparent [37]. One challenge is diagnosis, which requires recurrent evaluation based on fixed criteria [38,39]. In cases of premature rupture of membranes before 34 weeks, hospital admission, antibiotic prophylaxis, and antenatal corticosteroids are necessary. A potential explanation for the higher prevalence of AIS among foreign-born mothers could be limited access to or insufficient information about prenatal care and health services. In Gagnon et al.’s systematic review, 62.5% of studies reported worse antenatal care, none better, and 16.7% mixed results. There were delays in antenatal care but no differences at birth in a study from Belgium that examined obstetric care and perinatal complications between recent immigrants and long-term residents [40]. As we did not find any differences in the use of antenatal steroids, this explanation also seems unlikely in our study. A study conducted in Germany investigated the association between maternal migration status (first- and second-generation) and inflammation during pregnancy (after 24 weeks) by measuring plasma CRP and IL-6 levels [41]. There was a significant association between immigrant background and inflammation during pregnancy, which appeared to persist among second-generation immigrants. The biological pathway suggested by the authors involves chronic stress, which may lead to prolonged activation of the hypothalamic–pituitary adrenal axis [42]. This, in turn, may cause the resistance of glucocorticoid receptors in immune cells, resulting in higher levels of inflammation. Social deprivation, disadvantage, and perceived discrimination lead to chronic stress, which is a risk factor for inflammatory processes [43]. Experiences of perceived exclusion and discrimination, sometimes over generations, could be a particular risk for chronic stress and may explain increased levels of inflammation in the second generation of Turkish-origin women, for example.

The second effect of interest that remained after adjusting for confounding factors was an increased incidence of preeclampsia among mothers with higher LD to German, i.e., higher language barriers. Preeclampsia is a hypertensive disorder of pregnancy associated with increased perinatal mortality in both mother and child. In addition to hypertension after 20 weeks of gestation, there are new organ manifestations (cardiovascular, cerebral, haematological, pulmonary) that can lead to liver and kidney failure. Placental disorders, vascular changes, and immunological and inflammatory factors are thought to be the main pathophysiological drivers [44]. No genetic basis for the disease has yet been identified. The incidence of preeclampsia in immigrant women in Western countries varies greatly depending on maternal country of origin [45,46,47]. Furthermore, some studies suggest that the incidence of preeclampsia increases with the length of stay [48], pointing to a concept known as the immigrant paradox. This seems to contradict our results showing a higher rate of preeclampsia with higher LD. However, a Norwegian study showed that the reasons for migration can also lead to different results. In particular, labour migrants had lower adjusted odds of very preterm preeclampsia (OR 0.65), whereas refugees had higher adjusted odds (OR 1.41). Several factors must therefore be taken into account to explain the occurrence of preeclampsia including individual predisposition, behavioural, environmental, and health systems factors [49]. Overall, the sizes of the two fixed effects that survived adjustment for confounders are small. Moreover, we tested a range of associations but did not adjust for multiple testing.

The random effects included in our nested models showed small but stable variations in the associations between LD and perinatal health according to birth hospitals. Considering regional and organisational differences among hospital patient populations (e.g., percentage of immigrants), catchment areas (e.g., rural vs. urban, socio-economic distribution), and individual treatment experiences, this is not surprising. In addition, for instance, there are some German hospitals that provide relevant information such as printed leaflets in foreign languages and health care staff has translator services available at all times, while other hospitals may not offer such services. Nevertheless, the current data suggest that treatment disparities among hospitals in Germany may not be as severe as previously reported by US-based studies [50,51].

**Strengths and limitations.** The main strength of this multi-centre, prospective cohort study is the large sample size of mothers and infants born VP across all of Germany. The operationalisation of language barriers in the form of LD allowed us to break up the oversimplified categorisation of participants into “native vs. foreign-born”, and we controlled for known confounding variables such as multiple birth status, gestational age at birth, and infant biological sex.

This study also has several weaknesses. Despite its continuous scoring, LD was not normally distributed across the population. Because of funding constraints as well as timing limitations for data collection visits, the GNN sample participating in the 5–6-year follow-up only included ~30% of the originally enrolled infants, while mothers’ countries of birth and L1s were only assessed at that follow-up. Consequently, we were not able to compare the current sample’s characteristics to the originally enrolled participants, but an underestimation of the actual immigration-related disparities is most likely. Importantly, we did not assess infant mortality in this current study since all infants of the mothers participating in the follow-up survived. Moreover, follow-up assessments that qualified participants for inclusion in these current analyses were administered to participating parents in German only, not in immigrant families’ L1s. This likely created participation bias (due to a higher proportion of foreign-born mothers dropping out between birth and child age 5 years whose German language skills were not sufficient). Participating foreign-born mothers’ educational qualifications were only assessed according to the German educational system, so mothers who had obtained Abitur-equivalent degrees abroad were coded into a wrong category. In particular when working with immigrant groups, it is important to stress the contribution of the mother’s level of education. There is substantial overlap among vulnerabilities, creating intersectionality and additive risks for certain individuals [52]. Indeed, foreign-born mothers in the current sample were less likely than German mothers to have completed high school. Future studies should pay better attention to assessing immigrants’ educational and professional qualifications in a way that allows for globally comparable operationalisation, for instance, with the International Standard Classification of Education (ISCED) coding system [53]. Moreover, practitioners and policymakers need to start considering the broader implementation of translation services for immigrant families, for example, in conjunction with assessments such as the Migrant-Friendly Maternity Care Questionnaire [54] to facilitate immigrant-sensitive, equitable health care provision.

## 5. Conclusions

Our findings from this large, multi-centre, longitudinal cohort of VP-born infants suggest that maternal immigrant status and language barriers have a limited impact on mothers’ perinatal health and short-term neonatal outcomes (IVH and BPD) in Germany. After adjusting for potential confounding factors, the only associations were a higher incidence of AIS (among foreign-born mothers and their infants) and preeclampsia (among mothers with higher LD of their L1 to German). Taken together, based on the current data, this suggests that, regardless of immigrant background or language skills, there may be few inequalities in the perinatal health of pregnant women and their newborn very preterm babies in Germany. Nevertheless, this study only assessed short-term perinatal outcomes and does not allow for any conclusions about infants’ subsequent neurodevelopment.

## Figures and Tables

**Table 1:** Descriptive characteristics of the GNN sample by German vs. foreign-born mothers (*n* = 3606).

	Mother’s Country of Birth		
	German (*n* = 2687)	Foreign (*n* = 919)	*p*-Value
**Maternal characteristics**			
Mother’s age (*Mean* (*SD*), years)	31.47 (5.30)	32.20 (5.55)	<0.001
Mother’s educational level (*n* (%))			<0.001
High-school degree (Abitur)	1324 (51.0%)	298 (39.3%)	
No high-school degree	1273 (49.0%)	461 (60.7%)	
LD to German (*Mean* (*SD*); *Mdn*)	6.11 (22.68); 0.00	50.74 (46.81); 86.30	<0.001
Mode of delivery			0.186
Vaginal birth (*n* (%))	240 (8.9%)	94 (10.3%)	
Planned caesarean section (*n* (%))	2169 (80.9%)	712 (78.1%)	
Emergency caesarean section (*n* (%))	273 (10.2%)	106 (11.6%)	
Preeclampsia (*n* (%))	191 (7.1%)	74 (8.1%)	0.345
HELLP (*n* (%))	264 (9.8%)	61 (6.6%)	0.004
Complete lung maturity injections (*n* (%))	2459 (91.6%)	845 (91.9%)	0.730
**Infant characteristics**			
Sex (*n* (%) female)	1289 (48.0%)	449 (48.9%)	0.643
Gestational age at birth (*Mean* (*SD*), wks)	28.13 (2.39)	27.90 (2.54)	0.017
Birth weight (*Mean Fenton z-score* (*SD*))	−0.42 (0.91)	−0.35 (0.87)	0.057
Type of pregnancy (*n* (%) multiples)	1071 (39.9%)	330 (35.9%)	0.034
IVH (*n* (%))	472 (17.6%)	163 (17.7%)	0.907
BPD (*n* (%))	511 (19.0%)	181 (19.7%)	0.642
AIS (*n* (%))	557 (20.8%)	270 (29.4%)	<0.001
Sepsis (*n* (%))	352 (13.1%)	139 (15.1%)	0.122
Days in hospital (*Mean* (*SD*))	74.49 (34.22)	77.56 (36.24)	0.021

Abbreviations: SD = standard deviation; Mdn = median; HELLP = haemolysis elevated liver enzymes and low platelets; IVH = higher grade (>II) intraventricular haemorrhage; BPD = bronchopulmonary dysplasia; AIS = neonatal arterial ischemic stroke.

**Table 2:** Fully adjusted multilevel logistic mixed-effects models showing associations between LD and perinatal health.

*Dependent Variable* *OR (95% CI)*	Planned Caesarean Section (*n* = 2819)	Preeclampsia (*n* = 3152)	HELLP (*n* = 3151)	AIS (*n* = 3150)
***Fixed effects***				
LD (per SD)	1.00 (0.99, 1.00)	1.01 (1.00, 1.01) *	1.00 (0.99, 1.00)	1.00 (1.00, 1.00)
Mother foreign country of birth	1.00 (0.67, 1.46)	0.99 (0.67, 1.47)	0.74 (0.50, 1.11)	1.45 (1.13, 1.86) **
Mother’s education (high vs. low)	0.92 (0.70, 1.20)	0.94 (0.71, 1.24)	1.27 (0.99, 1.64)	0.98 (0.82, 1.18)
Mother’s age (per year)	1.00 (0.98, 1.03)	1.00 (0.98, 1.03)	1.00 (0.97, 1.02)	1.01 (1.00, 1.03)
Infant gestation at birth (per week)	1.21 (1.14, 1.28) ***	1.14 (1.08, 1.21) ***	1.12 (1.06, 1.18) ***	0.77 (0.74, 0.80) ***
Infant sex	1.30 (1.01, 1.69) *	1.44 (1.10, 1.90) **	1.37 (1.07, 1.75) *	0.95 (0.80, 1.14)
Multiple birth	2.17 (1.61, 2.91) ***	0.20 (0.14, 0.30) ***	0.18 (0.12, 0.25) ***	0.98 (0.81, 1.18)
Constant	0.03 (0.01, 0.21) ***	0.00 (0.00, 0.01) ***	0.00 (0.00, 0.03) ***	145.02 (41.704, 512.43) ***
***Random effects by birth hospital***				
SD (constant)	0.79 (0.59, 1.06)	0.23 (0.07, 0.72)	0.41 (0.24, 0.68)	0.40 (0.28, 0.56)
***Log-pseudolikelihood***	−856.06	−781.53	−904.25	−1535.26

Abbreviations: LD = linguistic distance; SD = standard deviation; HELLP = haemolysis elevated liver enzymes and low platelets; AIS = neonatal arterial ischemic stroke. Please note, for fixed effects, * = *p* < 0.05; ** = *p* < 0.01; *** = *p* < 0.001; significant effects of interest are marked in **bold**.

**Table 3:** Fully adjusted multilevel linear mixed-effects models showing associations between LD and VP infant duration of stay in hospital.

*Dependent Variable:*	Days in Hospital (*n* = 3152)
***Fixed effects, coefficient (95% confidence interval)***	
LD	0.01 (−0.02, 0.03)
Mother foreign country of birth	0.17 (−2.14, 2.48)
Mother’s education	1.11 (−0.50, 2.71)
Mother’s age	−0.12 (−0.26, 0.03)
Infant gestation at birth	−10.39 (−10.71, −10.06) ***
Infant sex	−0.36 (−1.91, 1.19)
Multiple birth	1.43 (−0.17, 3.03)
Constant	368.39 (357.47, 379.31) ***
***Random effects by birth hospital***	
SD (constant)	6.45 (5.01, 8.30)
***Log-pseudolikelihood***	−14,258.38

Abbreviations: LD = linguistic distance; VP = very preterm; SD = standard deviation. Please note, for fixed effects, *** = *p* < 0.001.

## Data Availability

Individual-level data are unavailable because of privacy or ethical restrictions in line with EU GDPR, but the data can be obtained from the corresponding author in aggregated form upon reasonable request.

## Appendix A

**Table A1:** List of languages spoken by families in the GNN sample including reported frequency (*n*) and linguistic distance (LD score) to German by German vs. foreign-born mothers (*n* = 3606).

Languages	German Mothers *n*	Foreign-Born Mothers *n*	LD Score
German	2444	304	0.00
Dutch	4	0	48.83
English	43	17	67.33
Danish	4	2	67.52
Swedish	0	1	69.79
Dutch and Portuguese ^1^	0	2	71.18
English and Russian ^1^	0	1	79.69
English and Spanish ^1^	2	0	80.16
Hindi and English ^1^	0	2	80.66
Greek and English ^1^	2	0	81.35
English and Twi ^1^	1	2	83.40
English and Turkish ^1^	1	0	83.55
Filipino	0	2	83.70
Italian	8	3	86.30
Romanian	1	3	88.17
Macedonian	0	6	88.94
Spanish and Italian	1	0	89.55
Portuguese and Italian ^1^	0	1	89.92
Greek and Italian ^1^	1	0	90.84
Serbian, Croatian ^2^	3	16	91.56
Russian and Serbian ^1^	0	1	91.80
Russian	2	117	92.04
Bulgarian	0	5	92.10
Dari Persian	1	5	92.89
Spanish	14	3	92.98
Portuguese	7	11	93.53
Hindi	0	1	94.00
Ukrainian	1	4	94.41
Greek	8	4	95.37
Albanian	0	24	95.68
French	5	7	95.73
French and Malagasy ^1^	0	3	95.78
Kurdish	1	11	96.71
Moroccan	3	6	96.74
Pashto	1	4	96.78
Polish and Turkish ^1^	1	0	98.14
Turkish and Kurdish ^1^	0	2	98.24
Hungarian	0	8	98.33
Greek and Arabic ^1^	2	0	98.89
Azerbaijani and Arabic ^1^	0	1	99.11
Twi	0	1	99.50
Tamil	0	6	99.67
French and Lingala ^1^	0	2	99.76
Turkish	50	52	99.77
Tamazight	0	1	100.48
Lebanese	0	1	101.32
Chinese	0	4	102.35
Arabic	15	20	102.40
Lingala	1	1	103.73
**Total *n***	2627	667	

^1^ If parents had indicated multiple L1s, these languages’ linguistic distance to German was averaged. ^2^ According to the Automated Similarity Judgement Program (ASJP) [30], these languages have the same linguistic distance to German; please note that some languages above may be considered part of the same language families, such as Moroccan with Arabic; however, there are nuanced differences between individual words that result in a slightly different linguistic distance to German according to the ASJP calculation.

## References

[1] Chawanpaiboon S., Vogel J.P., Moller A.-B., Lumbiganon P., Petzold M., Hogan D., Landoulsi S., Jampathong N., Kongwattanakul K., Laopaiboon M. (2019). Global, regional, and national estimates of levels of preterm birth in 2014: A systematic review and modelling analysis. Lancet Glob. Health.

[2] Lawn J.E., Ohuma E.O., Bradley E., Idueta L.S., Hazel E., Okwaraji Y.B., Erchick D.J., Yargawa J., Katz J., Lee A.C.C. (2023). Small babies, big risks: Global estimates of prevalence and mortality for vulnerable newborns to accelerate change and improve counting. Lancet.

[3] Ohuma E.O., Moller A.-B., Bradley E., Chakwera S., Hussain-Alkhateeb L., Lewin A., Okwaraji Y.B., Mahanani W.R., Johansson E.W., Lavin T. (2023). National, regional, and global estimates of preterm birth in 2020, with trends from 2010: A systematic analysis. Lancet.

[4] Marlow N., Ni Y., Lancaster R., Suonpera E., Bernardi M., Fahy A., Larsen J., Trickett J., Hurst J.R., Morris J. (2021). No change in neurodevelopment at 11 years after extremely preterm birth. Arch. Dis. Child.-Fetal Neonatal Ed..

[5] Cheong J.L., Anderson P.J., Burnett A.C., Roberts G., Davis N., Hickey L., Carse E., Doyle L.W. (2017). Changing neurodevelopment at 8 years in children born extremely preterm since the 1990s. Pediatrics.

[6] Sansavini A., Guarini A., Caselli M.C. (2011). Preterm Birth: Neuropsychological Profiles and Atypical Developmental Pathways. Dev. Disabil. Res. Rev..

[7] Zeitlin J., Szamotulska K., Drewniak N., Mohangoo A.D., Chalmers J., Sakkeus L., Irgens L., Gatt M., Gissler M., Blondel B. (2013). Preterm birth time trends in Europe: A study of 19 countries. BJOG.

[8] Pedersen G.S., Mortensen L.H., Gerster M., Rich-Edwards J., Andersen A.M. (2012). Preterm birth and birthweight-for-gestational age among immigrant women in Denmark 1978–2007: A nationwide registry study. Paediatr. Perinat. Epidemiol..

[9] Gagnon A.J., Zimbeck M., Zeitlin J., Alexander S., Blondel B., Buitendijk S., Desmeules M., Di Lallo D., Gagnon A., Gissler M. (2009). Migration to western industrialised countries and perinatal health: A systematic review. Soc. Sci. Med..

[10] Sigurdson K., Mitchell B., Liu J., Morton C., Gould J.B., Lee H.C., Capdarest-Arest N., Profit J. (2019). Racial/Ethnic Disparities in Neonatal Intensive Care: A Systematic Review. Pediatrics.

[11] Vidiella-Martin J., Been J.V. (2023). Maternal Migration Background and Mortality Among Infants Born Extremely Preterm. JAMA Netw. Open.

[12] Damsted Rasmussen T., Villadsen S.F., Kragh Andersen P., Smith Jervelund S., Nybo Andersen A.M. (2021). Social and ethnic disparities in stillbirth and infant death in Denmark, 2005–2016. Sci. Rep..

[13] Rasmussen T.D., Villadsen S.F., Andersen P.K., Clausen T.D., Nybo Andersen A.-M. (2019). Ethnic differences in the risk of caesarean section: A Danish population-based register study 2004–2015. BMC Pregnancy Childbirth.

[14] UN (2020). International Migrant Stock 2020.

[15] UN (2019). International Migrant Stock 2019.

[16] BMFSJ (2022). Gelebte Vielfalt: Familien mit Migrationshintergrund in Deutschland.

[17] Destatis Statistischer Bericht—Mikrozensus—Bevölkerung nach Migrationshintergrund—Erstergebnisse 2022 Statistisches_Bundesamt: 2023. https://www.destatis.de/DE/Themen/Gesellschaft-Umwelt/Bevoelkerung/Migration-Integration/Publikationen/Downloads-Migration/statistischer-bericht-migrationshintergrund-erst-2010220227005.html.

[18] Autor:innengruppe_Bildungsberichterstattung (2022). Bildung in Deutschland 2022.

[19] Clarke A., Isphording I.E. (2017). Language Barriers and Immigrant Health. Health Econ..

[20] Jaekel N., Ritter M., Jaekel J. (2023). Associations of students’ linguistic distance to the language of instruction and classroom composition with English reading and listening skills. Stud. Second. Lang. Acquis..

[21] NaDiRa (2023). Rassismus und seine Symptome. Bericht des Nationalen Diskriminierungs- und Rassismusmonitors.

[22] Palau M.A., Meier M.R., Brinton J.T., Hwang S.S., Roosevelt G.E., Parker T.A. (2019). The impact of parental primary language on communication in the neonatal intensive care unit. J. Perinatol..

[23] Eslier M., Deneux-Tharaux C., Schmitz T., Luton D., Mandelbrot L., Estellat C., Radjack R., Azria E. (2023). Association between language barrier and inadequate prenatal care utilization among migrant women in the PreCARE prospective cohort study. Eur. J. Public Health.

[24] Hamwi S., Barros H., Lorthe E. (2023). Migrant-Native Disparities in Obstetric Neuraxial Analgesia Use: The Role of Host-Country Language Proficiency. Anesth. Analg..

[25] Hamwi S., Lorthe E., Barros H. (2022). Host-country language proficiency and migrant-native disparities in prenatal care utilization: A nationwide study in Portugal. Birth.

[26] Dewalt D.A., Berkman N.D., Sheridan S., Lohr K.N., Pignone M.P. (2004). Literacy and health outcomes: A systematic review of the literature. J. Gen. Intern. Med..

[27] Abubakar I., Aldridge R.W., Devakumar D., Orcutt M., Burns R., Barreto M.L., Dhavan P., Fouad F.M., Groce N., Guo Y. (2018). The UCL-Lancet Commission on Migration and Health: The health of a world on the move. Lancet.

[28] Geisler I., Rausch T.K., Göpel W., Spiegler J. (2021). Extremely and very preterm-born children <1500 g show different weight development in childhood compared to their peers. Acta Paediatr..

[29] IOM (2019). International Migration Law: Glossary on Migration. International Organization for Migration (IOM). https://publications.iom.int/system/files/pdf/iml_34_glossary.pdf.

[30] Wichmann S., Brown C.H., Holman E.W. (2020). The ASJP Database.

[31] Levenshtein V. (1966). Binary codes capable of correcting deletions, insertions, and reversals. BibSonomy Cybern. Control Theory.

[32] Wichmann S., Holman E.W., Bakker D., Brown C.H. (2010). Evaluating linguistic distance measures. Phys. A Stat. Mech. Its Appl..

[33] Heslehurst N., Brown H., Pemu A., Coleman H., Rankin J. (2018). Perinatal health outcomes and care among asylum seekers and refugees: A systematic review of systematic reviews. BMC Med..

[34] (2005). Qualitätssicherungs-Richtlinie Früh- und Reifgeborene/QFR-RL. Anlage 3 (II.4.3 und II.5.1+ II.5.2). https://www.g-ba.de/downloads/62-492-3333/QFR-RL_2023-10-19_iK-2024-01-01_2024-01-19.pdf.

[35] Hüning B.M., Reimann M., Beerenberg U., Stein A., Schmidt A., Felderhoff-Müser U. (2012). Establishment of a family-centred care programme with follow-up home visits: Implications for clinical care and economic characteristics. Klin. Padiatr..

[36] Hüning B.M., Reimann M., Sahlmen S., Leibold S., Nabring J.C., Felderhoff-Müser U. (2016). Konzeptanalyse einer stationären und ambulanten psychosozialen familienzentrierten Betreuung in der Neonatologie in Zeiten des G-BA-Beschlusses. Klin. Padiatr..

[37] Combs C.A., Gravett M., Garite T.J., Hickok D.E., Lapidus J., Porreco R., Rael J., Grove T., Morgan T.K., Clewell W. (2014). Amniotic fluid infection, inflammation, and colonization in preterm labor with intact membranes. Am. J. Obstet. Gynecol..

[38] AWMF (2006). Empfehlungen zum Vorgehen Beim Vorzeitigen Blasensprung. https://register.awmf.org/assets/guidelines/015-029_S1_Empfehlungen_zum_Vorgehen_beim_vorzeitigen_Blasensprung_06-2006_06-2011_01.pdf.

[39] Maul H., Kunze M., Berger R. (2021). Current approach in preterm prelabor rupture of membranes: New definitions? Is CRP determination useful? Are alternatives in sight?. Gynakologe.

[40] Paquier L., Barlow P., Paesmans M., Rozenberg S. (2020). Do recent immigrants have similar obstetrical care and perinatal complications as long-term residents? A retrospective exploratory cohort study in Brussels. BMJ Open.

[41] Spallek J., Scholaske L., Duman E.A., Razum O., Entringer S. (2021). Association of maternal migrant background with inflammation during pregnancy—Results of a birth cohort study in Germany. Brain Behav. Immun..

[42] Cohen S., Janicki-Deverts D., Doyle W.J., Miller G.E., Frank E., Rabin B.S., Turner R.B. (2012). Chronic stress, glucocorticoid receptor resistance, inflammation, and disease risk. Proc. Natl. Acad. Sci. USA.

[43] Scholaske L., Brose A., Spallek J., Entringer S. (2019). Perceived discrimination and risk of preterm birth among Turkish immigrant women in Germany. Soc. Sci. Med..

[44] Chaiworapongsa T., Chaemsaithong P., Yeo L., Romero R. (2014). Pre-eclampsia part 1: Current understanding of its pathophysiology. Nat. Rev. Nephrol..

[45] Ray J.G., Wanigaratne S., Park A.L., Bartsch E., Dzakpasu S., Urquia M.L. (2016). Preterm preeclampsia in relation to country of birth. J. Perinatol..

[46] Urquia M.L., Glazier R.H., Gagnon A.J., Mortensen L.H., Nybo Andersen A.M., Janevic T., Guendelman S., Thornton D., Bolumar F., Río Sánchez I. (2014). Disparities in pre-eclampsia and eclampsia among immigrant women giving birth in six industrialised countries. BJOG.

[47] Naimy Z., Grytten J., Monkerud L., Eskild A. (2015). The prevalence of pre-eclampsia in migrant relative to native Norwegian women: A population-based study. BJOG.

[48] Nilsen R.M., Vik E.S., Rasmussen S.A., Small R., Moster D., Schytt E., Aasheim V. (2018). Preeclampsia by maternal reasons for immigration: A population-based study. BMC Pregnancy Childbirth.

[49] Johnson J.D., Louis J.M. (2022). Does race or ethnicity play a role in the origin, pathophysiology, and outcomes of preeclampsia? An expert review of the literature. Am. J. Obstet. Gynecol..

[50] Glazer K.B., Zeitlin J., Howell E.A. (2021). Intertwined disparities: Applying the maternal-infant dyad lens to advance perinatal health equity. Semin. Perinatol..

[51] Howell E.A., Zeitlin J. (2017). Improving hospital quality to reduce disparities in severe maternal morbidity and mortality. Semin. Perinatol..

[52] Bastia T., Datta K., Hujo K., Piper N., Walsham M. (2023). Reflections on intersectionality: A journey through the worlds of migration research, policy and advocacy. Gend. Place Cult..

[53] UNESCO (2012). International Standard Classification of Education. ISCED 2011.

[54] Gagnon A.J., DeBruyn R., Essén B., Gissler M., Heaman M., Jeambey Z., Korfker D., McCourt C., Roth C., Zeitlin J. (2014). Development of the Migrant Friendly Maternity Care Questionnaire (MFMCQ) for migrants to Western societies: An international Delphi consensus process. BMC Pregnancy Childbirth.

